# The MMSE should not be the sole indicator of fitness to drive in mild Alzheimer’s dementia

**DOI:** 10.1007/s13760-018-1036-3

**Published:** 2018-11-02

**Authors:** D. Piersma, A. B. M. Fuermaier, D. de Waard, P. P. De Deyn, R. J. Davidse, J. de Groot, M. J. A. Doumen, R. A. Bredewoud, R. Claesen, A. W. Lemstra, A. Vermeeren, R. Ponds, F. Verhey, W. H. Brouwer, O. Tucha

**Affiliations:** 1 Department of Clinical and Developmental Neuropsychology, University of Groningen, Groningen, The Netherlands; 2 Department of Neurology and Alzheimer Research Center, University Medical Center Groningen, Groningen, The Netherlands; 3 SWOV Institute for Road Safety Research, The Hague, The Netherlands; 4 CBR Dutch Driving Test Organisation, Rijswijk, The Netherlands; 5 Department of Neurology, Alzheimer Center, VU University Medical Center, Amsterdam, The Netherlands; 6 Department of Neuropsychology and Psychopharmacology, Maastricht University, Maastricht, The Netherlands; 7 Department of Psychiatry and Neuropsychology, School of Mental Health and Neurosciences (MHeNS), Maastricht University, Maastricht, The Netherlands

**Keywords:** Alzheimer’s dementia, Fitness to drive, Driving, MMSE

## Abstract

Since Alzheimer’s disease may affect driving performance, patients with Alzheimer’s disease are assessed on fitness to drive. On-road driving assessments are widely used, and attempts have also been made to develop strategies to assess fitness to drive in a clinical setting. Preferably, a first indication of fitness to drive is obtained quickly after diagnosis using a single test such as the Mini-Mental State Examination (MMSE). The aim of this study is to investigate whether the MMSE can be used to predict whether patients with Alzheimer’s disease will pass or fail an on-road driving assessment. Patients with Alzheimer’s disease (*n* = 81) participated in a comprehensive fitness-to-drive assessment which included the MMSE as well as an on-road driving assessment [PLoS One 11(2):e0149566, 2016]. MMSE cutoffs were applied as suggested by Versijpt and colleagues [Acta Neurol Belg 117(4):811–819, 2017]. All patients with Alzheimer’s disease who scored below the lower cutoff (MMSE ≤ 19) failed the on-road driving assessment. However, a third of the patients with Alzheimer’s disease who scored above the upper cutoff (MMSE ≥ 25) failed the on-road driving assessment as well. We conclude that the MMSE alone has insufficient predictive value to correctly identify fitness to drive in patients with very mild-to-mild Alzheimer’s disease implicating the need for comprehensive assessments to determine fitness to drive in a clinical setting.

## Introduction

In an ageing society, the number of patients with Alzheimer’s disease (AD) is increasing [1], and many of them drive a car when they receive their diagnosis. Continuation of driving after diagnosis is common [2, 3], and can be safe in the early stages of AD [4, 5]. However, with disease progression, driving performance will decline. Accordingly, there is consensus that patients with moderate-to-severe dementia should not drive anymore [4]. For patients with very mild-to-mild AD, individual assessments are necessary to determine their fitness to drive [6].

Despite the influence of AD on driving, the Belgian law on driving is rather vague in its referral to and instructions for AD, and does not implicate referral to an on-road driving assessment for all drivers with mild AD [7]. In The Netherlands, drivers who receive a diagnosis of AD have a moral obligation to report their diagnosis to the Dutch driving licence authority (CBR) [8]. Patients with very mild-to-mild AD (Clinical Dementia Rating < 2) are invited for an on-road driving assessment of approximately 45 min. CBR uses a protocol for patients with cognitive impairment to judge the driving performance of the patients. During the on-road driving assessment, patients drive in their own car with an expert of CBR in the passenger seat. CBR experts have long-lasting experience with on-road examinations and are trained to observe the effects of diseases on driving performance.

The major advantage of on-road driving assessments is the high validity, i.e., patients drive in the real world, but there are also disadvantages. On-road driving assessments are relatively costly, as compared to most neuropsychological tests. In addition, there is always a time gap between the clinical diagnosis and the on-road driving assessment. Patients may not report their diagnosis to the CBR instantly, and when they do it often takes several months until the on-road driving assessment is realised. Due to the rising number of patients with AD, it becomes increasingly difficult to assess all patients on the road early after diagnosis [9]. Meanwhile, it remains unclear whether patients with very mild-to-mild AD are safe to drive or not. On one hand, patients with AD have an increased accident risk [10] and should abstain from driving until their fitness to drive is determined. On the other hand, driving continuation serves the mobility needs of the patients with AD and having only limited recent driving experience decreases their chances of passing the on-road driving assessment [11].

Physicians diagnosing AD have to inform the patients about possible consequences for driving. Unsurprisingly, patients with AD and their family members often consult the treating physician for advice regarding driving [12], although physicians are usually not sufficiently equipped with proper tools to evaluate fitness to drive [6]. Unless available clinical tools could be used for this purpose, i.e., Versijpt and colleagues [7] recently suggested a framework using the Mini-Mental State Examination (MMSE) [13] as a starting point.

The MMSE is a screening instrument to acquire a global impression of cognitive functioning of a patient, and it is also used to stage the severity of AD [14]. The MMSE is easy to apply, because it can be administered with only little training, requires no technical or expensive equipment, and can be completed within 5–10 min. Patients with AD have to respond to verbally administered questions and instructions. The MMSE taps into various cognitive domains, including orientation, memory, and language. Since the MMSE is a short screening, cognitive domains of visuospatial abilities and executive functions are only assessed superficially [14, 15]. In brief, the MMSE is a practical, commonly used clinical tool, to assess global cognition.

The MMSE has also been used in studies about fitness to drive [6, 11, 16], in which a lower total MMSE score (range 0–30) was associated with a worse driving performance. Despite this association, research has shown repeatedly that the MMSE is not a useful instrument in predicting driving performance in mixed dementia samples [6, 17, 18]. Recently, Versijpt and colleagues [7] proposed MMSE cutoffs to trichotomize patients with AD specifically into temporarily safe, indeterminate, and unsafe drivers. The authors emphasise that many other factors are important for driving, and a final decision on driving cessation should only be made after thorough risk assessment. Nonetheless, the first step in their proposed guideline is to let the clinician provide a temporary indication of fitness to drive early after a diagnosis of AD using MMSE cutoffs. The aim of the current study is to evaluate the MMSE as a valid indicator of fitness to drive in patients with AD by taking MMSE scores and on-road driving assessments from a comprehensive fitness-to-drive assessment study [11]. MMSE cutoffs proposed by Versijpt and colleagues [7] will be used to classify patients with AD as safe or unsafe drivers. Patients with AD scoring below the lower cutoff (MMSE ≤ 19) are expected to fail the on-road driving assessment, whereas patients with AD scoring above the upper cutoff (MMSE ≥ 25) are expected to pass the on-road driving assessment.

## Methods

### Participants

Eighty-one patients with mild AD (Clinical Dementia Rating < 2) participated in this study. Their age ranged from 52 to 91 years (*M* = 72 years; SD = 9 years), and 53 (65%) were men. All patients were in possession of a valid driving licence and had a wish to continue driving. AD was diagnosed by a neurologist, geriatrician, psychiatrist, or general practitioner. Ten patients were additionally diagnosed with vascular dementia (VaD). Exclusion criteria were the diagnosis of other neurological or psychiatric conditions that may influence driving performance and usage of medications with a severe influence on driving ability.

### Measures

The MMSE [13, 19] is a screening instrument to acquire a global impression of cognitive functioning. It was administered as the first instrument in a comprehensive fitness-to-drive assessment in a clinical setting (see [11] for the full protocol). The sum score of the MMSE (range 0–30) was used.

The on-road driving assessment was performed in the patient’s own car on another day. These assessments were rated by approved experts on practical fitness to drive of CBR. They used a protocol for patients with cognitive impairments. Experts of CBR were blind to the patients’ MMSE scores. The CBR experts provided a pass, doubtful, or fail outcome after each on-road driving assessment. Patients with AD who passed the on-road driving assessment are regarded safe drivers, while patients who failed the on-road driving assessment are regarded unsafe drivers. The latter group would have lost their driving licence if their on-road driving assessment would have been part of an official relicensing procedure in The Netherlands. A doubtful outcome is not sufficient to renew a driving licence and is, therefore, regarded as a fail as well, but it indicates that it might be worthwhile to sign up for a second on-road driving assessment after adjusting the car (e.g., transition to automatic transmission) or additional driving lessons.

### Statistical analysis

A receiver operating characteristic (ROC) analysis was carried out to examine the accuracy of the MMSE in predicting fitness to drive (i.e., passing/failing the on-road driving assessment) of patients with AD. An ROC curve was created by plotting the sensitivity against the specificity. A larger area under the curve (AUC) indicates better predictive accuracy.

Patients with AD were trichotomized into three groups, i.e., unsafe, indeterminate, and safe, based on proposed MMSE cutoffs (≤ 19 and ≥ 25) [7]. The outcomes of the on-road driving assessments in these three groups were reported. Effects of adjustments of the MMSE cutoffs for the prediction of fitness to drive were described as well.

## Results

MMSE scores of the patients with AD (*n* = 81) ranged from 11 to 29 (*M* = 23.2, SD = 3.7). Thirty-five patients passed the on-road driving assessment, while 46 patients failed the on-road driving assessment (i.e., 41 fail and 5 doubtful outcomes combined). An ROC analysis revealed that the MMSE was significantly predictive for fitness to drive of patients with AD, with moderate predictive accuracy (AUC = 0.762, SE = 0.052, *p* < .001).

Patients with AD were divided into three groups as suggested by the MMSE cutoffs of Versijpt and colleagues [7] (Table 1). Thirteen patients had an MMSE score of 19 or lower, and all these patients (10 with AD, 3 with AD and VaD) failed the on-road driving assessment (Table 2). Thirty-three patients with an MMSE score between 20 and 24 were classified as indeterminate. Thirty-five patients had an MMSE score of 25 or higher, and 22 of them passed the on-road driving assessment (20 with AD, 2 with AD and VaD), 2 had a doubtful outcome (2 with AD), and 11 failed the on-road driving assessment (9 with AD, 2 with AD and VaD). This means that only 63% of the patients with AD scoring above the upper MMSE cutoff could renew their driving licence.

**Table 1:** Characteristics of the three groups of patients with Alzheimer’s dementia divided based on MMSE cutoffs

Characteristics	Group		
	MMSE ≤ 19 (*n* = 13)	MMSE 20–24 (*n* = 33)	MMSE ≥ 25 (*n* = 35)
Age [mean (SD)] (year)	76.9 (7.5)	71.9 (8.5)	69.7 (10.0)
Male sex [*n* (%)]	11 (84.6)	23 (69.7)	19 (54.3)
Education [mean of 7 stages (SD)]	4.1 (1.6)	4.9 (1.3)	5.2 (1.2)
CDR score [*n* (%)]			
0	0 (0.0%)	0 (0.0%)	1 (2.9)
0.5	6 (46.2)	29 (87.9)	32 (91.4)
1	7 (53.8)	4 (12.1)	2 (5.7)
MMSE score [mean (SD)]	16.9 (2.5)	22.2 (1.3)	26.4 (1.1)
Driving experience [mean (SD)] (y)	54.8 (8.1)^a^	50.2 (8.7)	46.3 (9.7)^b^

Education, Verhage scale for the Dutch educational level ranging from 1 (primary school not finished) to 7 (university level)

*CDR score* Clinical Dementia Rating total score, *MMSE score* Mini-Mental State Examination sum score (range 0–30)

^a^Data missing for one patient

^b^Data missing for two patients

**Table 2:** Application of MMSE cutoffs to predict fitness to drive on the road

MMSE scores	Classified as	Pass rate on-road driving assessment
≤ 19	Unsafe	0/13 (0%)
20–24	Indeterminate	13/33 (39%)
≥ 25	Safe	22/35 (63%)

*MMSE scores* Mini-Mental State Examination sum scores (range 0–30)

Adjusting the lower MMSE cutoff to 20 or lower resulted in an accurate prediction of failing the on-road driving assessment as well (0/16 patients with AD passed). However, adjusting the upper MMSE cutoff to an MMSE score of 26 or 27 and higher does not result in a more accurate prediction of fitness to drive compared to a cutoff of 25. For patients with AD with an MMSE score of exactly 27, the chance of passing the on-road driving assessment was only 50%. An even higher upper MMSE cutoff is not desirable, because the indeterminate group would become unreasonably large. Nonetheless, five out of the six patients with AD with an MMSE score of 28 or 29 passed the on-road driving assessment.

## Discussion

The aim of the current study was to investigate whether the MMSE alone could be used to accurately predict fitness to drive of patients with very mild-to-mild AD. The total score of the MMSE was significantly predictive for the outcome of an on-road driving assessment. Moreover, the lower MMSE cutoff (≤ 19) proposed in a framework by Versijpt and colleagues [7] accurately classified patients with AD as unsafe drivers. However, the higher MMSE cutoff (≥ 25) classified only 63% of the patients with AD correctly as safe drivers.

In a recent paper [20], consensus was sought among clinicians about various recommendations regarding driving and dementia. Clinicians agreed (> 90% agreement) that the MMSE and other tests have insufficient sensitivity or specificity to be used alone to determine fitness to drive in patients with dementia [20], which is in correspondence with the results from the current study and previous studies [6, 16, 18, 21]. Even though abnormal MMSE scores may indicate the need to further assess the driver at risk (> 90% agreement), clinicians did not fully agree (< 90% agreement) that substantial impairments as indicated by the MMSE, typically associated with moderate-to-severe dementia, may preclude safe driving. Various reasons were mentioned, including the lack of established cutoffs and the need to evaluate cognitive test scores in light of the full clinical picture (e.g., history, impairments in daily life, insight, and language problems) [20]. This view might change due to the accurate prediction of unsafe driving based on MMSE scores ≤ 19 in the current study. In this study, the group of patients with an MMSE ≤ 19 was rather small (*n* = 13) though; therefore, replication in a large sample of patients with AD is warranted. If the finding is replicated, patients with AD with an MMSE ≤ 19 could be advised not to take the on-road driving assessment, but to adapt to alternative transportation. In conclusion, MMSE scores ≤ 19 clearly appear suspicious of unsafe driving, corresponding to high levels of sensitivity of the MMSE for moderate-to-severe levels of dementia [14, 22]—stages of dementia, where driving is generally strongly discouraged [4, 20].

The possibility of using an upper MMSE cutoff (e.g., ≥ 25) for the prediction of fitness to drive in patients with AD should be questioned. An accurate prediction of passing the on-road driving assessment for only 63% of the patients with AD with an MMSE score ≥ 25 is only slightly better than an estimation by chance (i.e., randomly guessing which 50% of patients with AD will pass the on-road driving assessment). One should be very careful in arguing that it can be temporarily advised to drive for patients with AD based on an MMSE score ≥ 25, which was proposed by Versijpt and colleagues [7]. The MMSE is a short screening for global cognitive functioning, which may insufficiently cover several cognitive domains that are highly relevant for driving, namely visuospatial abilities and executive functions [14, 15, 23, 24]. Patients with cognitive impairments in these domains may be unsafe drivers, even when having high MMSE scores due to relatively preserved orientation and language functions. Consequently, a score above the upper MMSE cutoff should not be used as the sole indicator of fitness to drive in AD.

Other limitations of the MMSE include that scores are affected by age, education, and cultural background [14]. At high age (> 75–80 years), MMSE scores are usually lower risking an overestimation of the severity of cognitive impairment. Furthermore, results of the MMSE should be interpreted with caution for patients with a very low education (e.g., patients who cannot read), as they might have a lower total MMSE score for this reason. At the other end of the spectrum, patients with a very high education may be able to mask their cognitive impairment and have high total MMSE scores despite cognitive impairments [14]. The MMSE is highly demanding on verbal functions; therefore, it is crucial that the patient understands and speaks the language well in which the MMSE is conducted. In conclusion, MMSE scores should never be interpreted on their own, but always in combination with at least demographic and further clinical information about the patient with AD.

In the context of fitness to drive, the current results suggest that an MMSE score of 19 or lower might be sufficient reason to advise patients with AD to not drive until their fitness to drive is investigated further as these patients are likely to fail an on-road driving assessment. For exceptional patients with AD with an MMSE score ≤ 19 who are still safe to drive, the risk of premature driving cessation remains limited when the final decision is only made after thorough risk assessment. In contrast, the results of this study do not support the use of an upper MMSE cutoff to state that it is temporarily allowable for patients with AD with high MMSE scores to continue driving, because many patients with AD with MMSE scores of 25 and higher were unable to pass an on-road driving assessment. The risk of advising continued unsafe driving appears to be high. This implicates that, even when MMSE scores are high, fitness to drive of patients with AD should be critically evaluated [18]. Even though pass rates on the on-road driving assessments of the three groups with high, intermediate, and low MMSE scores may be informative for patients with AD and their family members, an individual high MMSE score should not be used as the sole indicator of fitness to drive. To predict fitness to drive of patients with mild AD in a clinical setting, comprehensive fitness-to-drive assessments are necessary [6, 11].

## Data Availability

The data set analysed during the current study is available in the DataverseNL repository, http://hdl.handle.net/10411/20673. Further details may be obtained from the University of Groningen Research Data Office: researchdata@rug.nl.
